# The role of focus back effort in the relationships among motivation, interest, and mind wandering: an individual difference perspective

**DOI:** 10.1186/s41235-023-00502-0

**Published:** 2023-07-13

**Authors:** Hong He, Yunyun Chen, Ting Li, Hui Li, Xuemin Zhang

**Affiliations:** 1 Institute of Brain and Psychological Sciences, Sichuan Normal University, Chengdu, China; 2 Beijing Key Lab of Applied Experimental Psychology, Faculty of Psychology, Beijing Normal University, Beijing, China; 3 State Key Laboratory of Cognitive Neuroscience and Learning & McGovern Institute for Brain Research, Beijing Normal University, Beijing, China

**Keywords:** Mind wandering, Focus back effort, Motivation, Interest, Individual difference

## Abstract

Considerable research suggests a link between mind wandering and diminished levels of motivation and interest. During episodes of mind wandering, individuals may engage in efforts to redirect their attention back to the task at hand (known as focus back effort). Building on the resource-control hypothesis, we hypothesized that the influence of interest and motivation on mind wandering may be mediated by focus back effort. In Study 1, we employed a latent-variable approach to investigate these relationships across three tasks with varying cognitive demands. The results showed that individual differences in interest indirectly influenced mind wandering through the mediating factors of motivation and focus back effort. Furthermore, individual differences in interest indirectly predicted task performance through the mediating factors of motivation, focus back effort, and mind wandering during the high-load task. In Study 2, we replicated the relationships among these factors in a reading comprehension task. The results consistently support the role of focus back effort as an adaptive mechanism for executive control, enabling the allocation of cognitive resources to both mind wandering and task performance. These findings underscore the significance of focus back effort in elucidating the interplay between mind wandering, motivation, interest, and task performance. Importantly, our results align with the resource-control theory.

**Supplementary Information:**

The online version contains supplementary material available at 10.1186/s41235-023-00502-0.

## Significance

It is a common experience for individuals to begin a task, such as reading a scholarly article, and later realize that their minds have wandered to different topics. Mind wandering occurs for approximately one-third of the time individuals are awake, and it is widely acknowledged to have a detrimental impact on task performance. Therefore, investigating the factors that influence mind wandering is essential to mitigate its negative effects and improve task performance. The implications of these findings are of great significance in the realms of education and the workplace. Previous studies have suggested that individual differences in interest could predict variations in motivation, which, in turn, can impact mind wandering and ultimately affect task performance. In light of this, we conducted two studies, and the results revealed that individuals with higher levels of interest and motivation were more likely to invest additional effort in redirecting their attention back to the task at hand, resulting in a reduction in the frequency of mind wandering episodes. This reduction in mind wandering had a positive influence on task performance, both in tasks commonly employed to study mind wandering and in a reading comprehension task. These findings imply that individuals regulate the allocation of their cognitive resources during tasks under the influence of their interest and motivation, thereby influencing mind wandering and task performance.

## Introduction

While attending a class lecture or engaged in a reading process, an individual’s attention may shift from the course or reading material to unrelated topics, such as planning a weekend camping trip or deciding what to eat for dinner. This phenomenon is known as mind wandering, wherein an individual's thoughts shift away from the primary task and instead focus on internal information unrelated to the task at hand (Smallwood & Schooler, 2006). Humans spend at least one-third of their waking hours lost in thoughts unrelated to their ongoing activities (Kane et al., 2017). Consequently, there is a growing interest among researchers in investigating the factors that influence mind wandering. Previous research has indicated that an individual's interest and motivation in a given task can influence the propensity to experience mind wandering. For example, individuals who possess a strong interest in the course material or the reading text are more likely to be motivated to maintain their attention, resulting in reduced mind wandering and improved performance on course-related assessments and reading comprehension tasks (Seli et al., ; Soemer & Schiefele, 2019; Unsworth & McMillan, 2013). However, an unexplored question is whether interest and motivation influence mind wandering by regulating an individual's allocation of attentional resources. This inquiry prompts us to consider whether individuals with higher levels of interest and motivation invest more effort in redirecting their attention back to the task, ultimately leading to a reduction in mind wandering.

Focus back effort is defined as the effort of trying to refocus on the current activity while in a mind wandering state (He et al., 2021a, 2021b; He et al., 2021a, 2021b). Its assessment involves randomly asking participant during a task to indicate the extent to which they were attempting to redirect their attention back to the task (He et al., 2021a, 2021b; He et al., 2021a, 2021b). In our prior investigation, we observed a positive correlation between focus back effort and functional connectivity within the nodes of the executive network (He et al., 2021a, 2021b). This finding aligns with a theoretical model that proposes a close relationship between the experience of effort and executive control (Kurzban et al., 2013). Additionally, a previous study demonstrated a negative association between focus back effort and mind wandering in a laboratory setting (He et al., 2021a, 2021b), indicating that individuals who put more effort into refocusing on the task are less likely to experience mind wandering. These findings imply that focus back effort may reflect the adjustment of executive control in regulating cognitive resources between mind wandering and the task at hand.

In addition to investigating the relationship between focus back effort and mind wandering, numerous studies have provided evidence supporting the notion that motivation plays a significant role in decreasing the frequency of mind wandering (Antrobus et al., 1966; Frank et al., 2015; Robison & Unsworth, 2018; Seli et al., 2015, 2018; Smallwood et al., 2007; Unsworth & McMillan, 2013). For instance, Frank et al (2015) discovered a negative association between self-reported mind wandering and levels of motivation to excel in a reading comprehension task. Moreover, informing participants that they have the opportunity to end the experiment early if they perform well has been demonstrated to enhance their motivation to excel in the focal task and subsequently lead to a reduction in mind wandering (Seli et al., 2017a, 2017b). Furthermore, a multifaceted approach integrating cognitive, dispositional, and contextual predictors has established a significant association between individual differences in mind wandering and motivation (Robison et al., 2020). Specifically, Robison et al. (2020) consistently found a connection between mind wandering and motivation across various tasks. Collectively, these results indicate that individuals with greater task-oriented motivation are less prone to experiencing mind wandering during the execution of said task.

Motivational factors have consistently been demonstrated to be associated with interest, suggesting that individuals who possess a higher level of interest are more motivated to perform a given task (Hidi & Harackiewicz, 2000). Previous studies have provided empirical supports for the relationship between individual differences in mind wandering and interest. Specifically, task interest and mind wandering are negatively related to each other (Grodsky & Giambra, 1990; Jackson & Balota, 2012; Kahmann et al., 2022; Robison & Unsworth, 2018; Smallwood et al., 2009a, 2009b). For example, individual differences in interest have been found to be linked to mind wandering in educational contexts (Unsworth & Mcmillan, 2017). To gain further insight into the interplay among motivation, interest, and mind wandering, Unsworth et al. (2013) conducted a study focusing on individual differences. Their findings revealed that motivation served as a mediating factor in the relationship between interest and mind wandering in a reading comprehension task. Unsworth et al. (2013) treated motivation and interest as domain-specific factors.

Another potential factor that may contribute to the relationship between mind wandering and domain-specific variables, such as interest and motivation, is focus back effort. Notably, findings from a study on mood induction suggest that domain-specific factors, such as mood, can influence an individual's level of focus back effort (He et al., 2021a, 2021b). Taking it a step further, it is reasonable to propose that other domain-specific factors, including interest and motivation, could also have an impact on focus back effort. For instance, when an individual is engaged in a task that he or she finds interesting and is highly motivated to excel in, the individual is likely to exert considerable effort in redirecting attention to the task whenever mind wandering occurs. Therefore, we hypothesized that individual differences in interest predict levels of motivation, which in turn predict the degree of focus back effort, ultimately resulting in variations in mind wandering experiences. This hypothesis is supported by the demonstration that motivation plays a role in reallocating resources applied to executive functions, thereby influencing processes that share cognitive resources with the targeted functions (Pessoa, 2009).

Two hypotheses that appear to be in conflict with each other have been put forth regarding the relationship between individual ability in executive processes and mind wandering. According to the attentional resource hypothesis, mind wandering consumes the same attentional resources required for the primary task (Smallwood & Schooler, 2006). Consequently, as the attention demands of a specific task increase, the portion of available resources allocated to mind wandering decreases. In contrast, the control failure hypothesis proposes that whenever control processes fail, the primary task goal may be replaced by task-unrelated goal representations, triggering mind wandering (Mcvay & Kane, 2010). Both hypotheses are supported by empirical evidence. For example, individuals tend to exhibit less mind wandering in more demanding tasks, supporting the attentional resource hypothesis (Forster & Lavie, 2009). Conversely, mind wandering is negatively associated with task performance in attentional control tasks (Randall et al., 2014), supporting the control failure hypothesis that mind wandering can be interpreted as a temporary interruption or interference with executive control processes. However, both hypotheses treat the executive function as a fixed individual ability and fail to fully explain the observed increase in mind wandering and decline in task performance over time. To address this, Thomson et al., (2015a, 2015b) argue that the attentional resource hypothesis and the control failure hypothesis are complementary and propose that their combination provides a more comprehensive explanation of mind wandering. Thomson et al., (2015a, 2015b) introduce the resource-control theory of mind wandering, which posits that mind wandering is the default state of human beings. They suggest that there is a continuous bias for resources, which is fixed and competed for by mind wandering and on-task thought, to be directed towards mind wandering. According to the resource-control theory, as the time on task increases, the executive control needed to maintain an active goal and suppress mind wandering decreases (Thomson et al., 2015a). Subsequent studies have provided further support for this theory, showing that time on task is positively correlated with mind wandering and is negatively associated with task performance (Brosowsky et al., 2020; Krimsky et al., 2017).

According to the resource-control hypothesis, executive control can be modulated by individuals depending on the context, and the decline in motivation may contribute to the fading of executive control over time. In other words, motivation may influence the allocation of executive resources, preventing mind wandering from consuming the resources necessary for the task at hand. However, no empirical study to date has yet investigated this relationship. As we hypothesized that focus back effort reflects the adjustment of executive control in the resource-control theory, it is expected that individuals who are highly motivated to complete a task will exhibit a higher level of focus back effort, leading to a reduction in mind wandering episodes.

Mind wandering can impose costs on task performance as attention becomes decoupled from external stimuli (Mooneyham & Schooler, 2013; Smallwood & Schooler, 2006). According to the attentional resource hypothesis (Smallwood & Schooler, 2006), mind wandering utilizes the same attentional resources required for the primary task and this idea is supported by various findings. For instance, studies have shown no significant relationship between mind wandering and error rates in low-load sustained attention to response tasks (SART) (Baird et al., 2011; Ruby et al., 2013). Additionally, researchers measured mind wandering across tasks with varying levels of cognitive load and observed that under demanding conditions, mind wandering has a more pronounced and noticeable negative impact on task performance (Greve & Was, 2022; Rummel & Boywitt, 2014). This phenomenon can also be explained by the more recent resource-control theory (Thomson et al., 2015a), which proposes that both task-related thoughts and mind wandering compete for a finite pool of cognitive resources, with allocation depending on task demands. Motivated by the findings of Unsworth and McMillan (2013) and the aforementioned hypotheses (Pessoa, 2009; Smallwood & Schooler, 2006; Thomson et al., 2015a), we hypothesized that motivation, focus back effort, and mind wandering mediate the relationship between interest and performance in high-load tasks, but not in low-load tasks.

Our goal is to investigate the role of focus back effort in the relationships among interest, motivation, and mind wandering. To provide the theoretical foundation, we aimed to explore whether focus back effort aligns with the adjustment of executive control proposed by the resource-control theory. We hypothesize that focus back effort reflects the adjustment of executive control in the resource-control theory, with the expectation that it will decrease over time during a task and be influenced by an individual's evaluation of the task's difficulty. In other words, we propose that the level of focus gradually diminishes over time (pre-H1), and the level of focus back effort increases with the task's level of challenge (pre-H2). We then addressed two main research questions. First, how do interest, motivation, and focus back effort influence mind wandering? Our first hypothesis is that individual differences in interest predict motivation, which predicts focus back effort, which in turn predicts mind wandering (H1). Second, how do interest, motivation, focus back effort, and mind wandering influence task performance? We hypothesized that in the high-load task (but not in the low-load task), individual differences in interest predict motivation, which further predicts focus back effort. Focus back effort, in turn, predicts mind wandering, which ultimately influences task performance (H2). To address the research questions, we conducted investigations focusing on individual differences.

## Study 1

In Study 1, participants completed three basic laboratory tasks (i.e., SART, 0-back task, 1-back task). Thought probes were inserted within the tasks to sample participants' mind wandering states and assess their levels of focus back effort. The SART was selected for Study 1 due to its widespread use in mind wandering research (Gyurkovics et al., 2020; He et al., 2021a, 2021b; Mcvay & Kane, 2009, 2012a; Robertson et al., 1997; Smallwood et al., 2004; Unsworth & McMillan, 2014). Furthermore, low- and high-load tasks, such as N-back tasks, are frequently utilized by mind wandering researchers to examine the demand-sensitive relationship between mind wandering and other variables (Ju & Lien, 2018; Robison et al., 2020; Rummel & Boywitt, 2014). By employing tasks with different loads and the hierarchical linear modeling (HLM) approach, we aimed to provide support for the notion that focus back effort reflects the regulation of executive control in the resource-control theory (pre-H1 and pre-H2). Additionally, confirmatory factor analysis (CFA), structural equation models (SEMs), and mediation models were employed to test the relationships among interest, motivation, focus back effort, mind wandering, and task performance (H1 and H2).

### Methods

#### Participants

The participants of this study were derived from an ongoing project that aimed to investigate the characteristics of focus back effort. A total of 212 university students completed both N-back tasks and the SART. The sample size was determined to include data from as many participants as possible over two complete terms. Data from 5 participants were excluded because they did not report mind wandering in any of the three tasks (i.e., SART, 0-back, 1-back), resulting in a final sample of 207 participants aged 17–29 years (male: n = 56, female: n = 151, mean age = 21.02, *SD* = 2.47). A sample size of 207 participants was considered sufficient for the study, as typically 10 samples are required to estimate a single parameter in SEM (Grace, 2006), and a sample size of 150 was recommended. Each participant was tested individually after providing a signed consent form (written informed consent was provided by legal guardians for participants who were under 18 years old). The Ethics Committee in China approved the study.

#### Procedure

After signing the consent form, each participant completed tasks in the laboratory. The modified 1-back task and modified 0-back tasks were performed in a counterbalanced order, with the SART administered after the N-back tasks. Each task lasted about 15 min. Following task completion, participants were asked to complete a series of questionnaires, which were not analyzed in the present study. Before each formal task, participants received instructions and engaged in practice. They were instructed to practice until they reached a target accuracy of 75% during each practice session.

#### Apparatus

The stimuli were displayed on a 17-inch CRT monitor with a resolution of 1024 pixels × 768 pixels and an 85 Hz refresh rate. The tasks were performed using the Psychtoolbox extension in MATLAB R2019a. Participants were positioned approximately 60 cm away from the screen.

#### Tasks

*Sustained attention to response task* The SART is a Go/No Go task that is sensitive to mind wandering occurrences (Gyurkovics et al., 2020; Mcvay & Kane, 2009; Smallwood et al., 2004). The current version of the SART largely replicated the SART adopted by Gyurkovics et al. (2020) and has been used in a previous study (He et al., 2021a, 2021b). In the Go/No Go task, digits 1 through 9 were randomly presented one at a time in white on a black screen. Each digit appeared for 1250 ms, followed by a 1250 ms blank black screen. The duration of the number's visibility on the screen and the interval between each number's appearance were determined based on previous research (Gyurkovics et al., 2020; Mittner et al., 2014). The SART consisted of two blocks of 131 trials, with a short self-paced break between the blocks. For digits 1 through 9 (except for 3) (Go stimuli, 85.5% of the 262 trials), participants were instructed to press the space bar. For digit 3 (No Go stimuli, 10.69% of trials), participants were instructed to withhold their responses. The remaining trials (3.82%) were thought probes, asking participants to characterize their thoughts preceding the probe. Furthermore, the No Go stimulus was never immediately preceded or followed by a thought probe or another No Go stimulus. The digits were in Courier New font and shown in one of five possible font sizes (120, 100, 94, 72, and 48 points), aligning with previous SART studies (Mittner et al., 2014; Nayda & Takarangi, 2021; Seli et al., 2016a, 2016b, 2016c; 2017a, 2017b; Thomson et al., 2015a, 2015b). Participants completed a practice session consisting of 20 trials and 1 thought probe.

*Modified N-back tasks* The participants performed two modified N-back tasks adapted from Ju and Lien (2018). In both tasks, digit sequences (1–9) were presented one digit at a time on a black background, either in red (target) or white (non-target) color. For the red digit, participants were instructed to press a key (either the left or right arrow button on the keyboard) to identify whether the digit was even or odd in the 0-back task and whether the digit shown directly before the target was even or odd in the 1-back task. For the white digit, participants were instructed not to press a key in either task. In both tasks, each digit had an equal probability of occurrence, with the red digit appearing in 20% of the 275 trials and the white digit appearing in 80% of the trials. The stimuli were presented in Courier New font size 72. In each of the N-back tasks, the digit appeared on a black background for 1500 ms, followed by a 1000 ms blank black screen. The participants completed 10 blocks in both the 0-back and 1-back tasks, with each block containing a different number of trials (10, 15, 18, 20, 25, 30, 35, 37, 40, and 45). Thought probes appeared at the end of each block. The sequence of blocks was randomly arranged, with the following restrictions on trial sequence: (a) no target was shown in the last five trials of one block, (b) no more than two targets were consecutively shown, and (c) the first trial of each block was the non-target. The practice phase of each N-back task comprised 20 trials and one thought probe.

*Thought probes* To measure the participants’ ongoing thoughts, experience sampling methodology (ESM) (Smallwood & Schooler, 2006) was used in the SART and N-back tasks. The probe-caught protocol consisted of two questions that sampled the participants’ ongoing experiences:

(1) Just prior to being asked, was your attention on- or off-task? (1–completely on-task, 6–completely off-task).

(2) To what extent were you trying to focus back on the task? (1-not at all, 6-very much).

When these questions appeared, participants rated their experiences on a continuous scale using the computer mouse. Participants could choose the following responses to the first question: 1 is considered that participants were completely on task, whereas 2–6 are considered that their minds had wandered to some extent (Bertossi & Ciaramelli, 2016). Participants were informed that during the experience of mind wandering, they might try to redirect their attention back to the task. Participants were required to select an option for the second question based on their own actual experience. To limit the response bias, question 2 appeared regardless of the participant’s answers in question 1. This approach has been used in previous studies (Christoff et al., 2009; Karapanagiotidis et al., 2017).

*Interest and motivation* Each participant evaluated his or her interest in the content of the task on a six-point scale ranging from 1 to 6, followed by a question sampling the motivation to perform well on a six-point scale ranging from 1 to 6 at the end of each task. Participants who scored higher on interest and motivation were considered more interested in the task and more motivated to perform the task. The participants provided ratings for interest and motivation questions via the computer mouse.

#### Statistical analysis

The HLM approach was employed to explore whether focus back effort decreases over time (see Additional file 1). Consistent with prior research (Mills et al., 2018; Smith et al., 2018; Tusche et al., 2014), the mind wandering score for each task was calculated as the average rating of the first question. The focus back effort score for each participant was determined by averaging the scores of all responses to question 2 when the answer to question 1 ranged from 2 to 6. We then labeled the mind wandering score in the SART as MW-SART, in the 0-back task as MW-0B, and in the 1-back task as MW-1B. Similarly, we labeled the focus back effort score in the SART as FBE-SART, in the 0-back task as FBE-0B, and in the 1-back task as FBE-1B. We also labeled interest and motivation using the same criteria: Interest-SART, Interest-0B, Interest-1B, Motivation-SART, Motivation-0B, and Motivation-1B. Furthermore, the omission error was calculated for the SART, which serves as an indicator of sustained attention and has been associated with mind wandering (Cheyne et al., 2009). When examining the relationship between task performance and mind wandering, we considered the percentage of omission errors in the SART and the percentage of errors in the N-back tasks.

Descriptive and correlation analyses were performed using IBM SPSS 19.0, while CFA, SEM, and mediation analyses were performed in Amos 19.0. Initially, CFA was used to examine the construct structure of the data. Subsequently, several SEMs were developed to address the first research question. In the CFA and SEMs, we included MW-SART, MW-0B, MW-1B, FBE-SART, FBE-0B, FBE-1B, Motivation-SART, Motivation-0B, Motivation-1B, Interest-SART, Interest-0B, and Interest-1B as manifest variables. Each measure was constrained to load exclusively on its corresponding primary factor. Model fit was evaluated via the following fit indices (Kline, 1998): chi-square statistics (χ^2^), root mean square error of approximation (RMSEA, less than 0.08), standardized root mean residual (SRMR, less than 0.08), non-normed fit index (NNFI, greater than 0.90), comparative fit index (CFI, greater than 0.90), and Akaike information criterion (AIC). Mediation models were tested using Amos with 1000 bootstrap samples and a 95% confidence interval (CI). The indirect effect was deemed significant when the CI did not include 0 (Preacher & Hayes, 2008). In addition, the sequential goodness of fit (SGoF) method was applied to control for family-wise error in all indirect paths (Carvajal-Rodríguez et al., 2009).

### Results and discussion

#### Descriptive statistics

The descriptive statistics for variables in Study 1 can be found in Additional file 1: Table S1. The results indicated that when participants reported being completely focused on the task in the first question, their responses to the second question indicated that they made no attempt to redirect their attention back to the task. The results revealed that all variables were either normally distributed or transformed to achieve normality. The findings from the HLMs, presented in Additional file 1: Tables S2, S3, and S4, showed a decline in focus back effort across trials in all three tasks. These findings support the view that focus back effort may reflect the executive control adjustment that fades over time, which is required to suppress mind wandering according to the resource-control theory (Thomson et al., 2015a). The Pearson correlation coefficients for all measures can be found in Additional file 1: Table S5. Furthermore, Additional file 1: Table S6 displays the proportions of participants with different responses regarding focus back effort and motivation in the tasks. Notably, some participants reported high focus back effort but low motivation and some individuals indicated low focus back effort but high motivation.

Moreover, the error rate in the 1-back task was significantly higher than that in the 0-back task (*t* = 9.666, *p* < 0.001, Cohen’s *d* = 0.671), while the mind wandering score in the 1-back task was lower than that in the 0-back task (*t* = − 6.385, *p* < 0.001, Cohen’s *d* = 0.444), indicating successful manipulation of task load. Additionally, motivation in the 1-back task was higher than that in the 0-back task (*t* = 6.488, *p* < 0.001, Cohen’s *d* = 0.451), which aligns with the "difficulty law of motivation" proposed by Hillgruber (1912). This law posits that task difficulty influences the motivation for expending effort and cognitive control (Hillgruber, 1912). Furthermore, the significant difference in focus back effort between the 0-back and 1-back tasks (*t* = 2.140, *p* = 0.034, Cohen’s *d* = 0.149) is in line with the resource-control hypothesis (Thomson et al., 2015a), indicating that individuals can adapt executive control based on contextual demands.

#### Confirmatory factor analysis

**Fig. 1:**
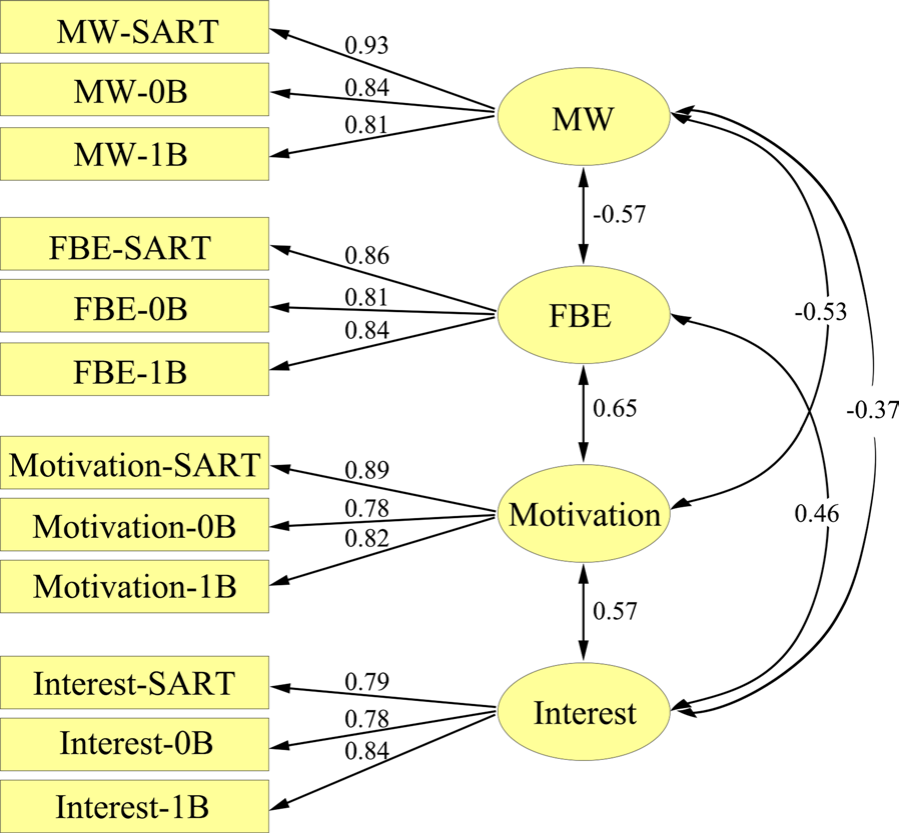
Confirmatory factor analysis (CFA) for mind wandering (MW), focus back effort (FBE), motivation, and interest. The correlations between the latent variables (circles) are represented by double-headed arrows linking these variables. Single-headed arrows from latent variables to observed variables (squares) represent the loadings of each manifest variable onto the latent variable. All numbers appearing beside each arrow are standardized. All paths are statistically significant at the *p* < 0.05 level. MW-SART = mind wandering in the sustained attention to response task; MW-0B = mind wandering in the 0-back task; MW-1B = mind wandering in the 1-back task; FBE-SART = focus back effort in the sustained attention to response task; FBE-0B = focus back effort in the 0-back task; FBE-1B = focus back effort in the 1-back task; Motivation-SART = motivation in the sustained attention to response task; Motivation-0B = motivation in the 0-back task; Motivation-1B = motivation in the 1-back task; Interest-SART = interest in the sustained attention to response task; Interest-0B = interest in the 0-back task; Interest-1B = interest in the 1-back task

CFA was conducted to determine the data structure for mind wandering (MW-SART, MW-0B, MW-1B), focus back effort (FBE-SART, FBE-0B, FBE-1B), motivation (Motivation-SART, Motivation-0B, Motivation-1B), and interest (Interest-SART, Interest-0B, Interest-1B), as depicted in Fig. 1. Three distinct mind wandering parcels were constructed: MW-SART, MW-0B, and MW-1B, representing the mind wandering scores obtained during the SART, 0-back task, and 1-back task, respectively. These parcels were then used to create a mind wandering factor by loading them onto the same factor. Similarly, separate factors were created for interest and motivation by loading the corresponding scores from the three tasks onto their respective factors. All factors were allowed to correlate, and each measure exclusively loaded onto the primary factor of interest. These measures showed acceptable reliabilities, with Cronbach’s alpha values of 0.893 for mind wandering, 0.868 for focus back effort, 0.867 for motivation, and 0.843 for interest. The fit of this model was acceptable, *χ*^2^ (48) = 103.38, RMSEA = 0.07, SRMR = 0.04, NNFI = 0.95, CFI = 0.96, AIC = 163.38, suggesting that the model provided an adequate characterization of the data pattern. The measures were observed to be stable indicators of individual differences, with variables from each task significantly loading on their respective constructs (*ps* < 0.001). Consistent with our prior research, mind wandering exhibited a significant correlation with focus back effort (He et al., 2021a, 2021b). Furthermore, the results indicated significant relationships between motivation, interest, and mind wandering, aligning with previous findings (Unsworth & McMillan, 2013). Specifically, focus back effort and motivation displayed strong correlations with mind wandering. Given the robust associations between interest and focus back effort, between interest and motivation, and the moderate relationship between interest and mind wandering, it is plausible that interest may predict mind wandering through the mediating factors of motivation and focus back effort.

#### Structural equation models

**Fig. 2:**
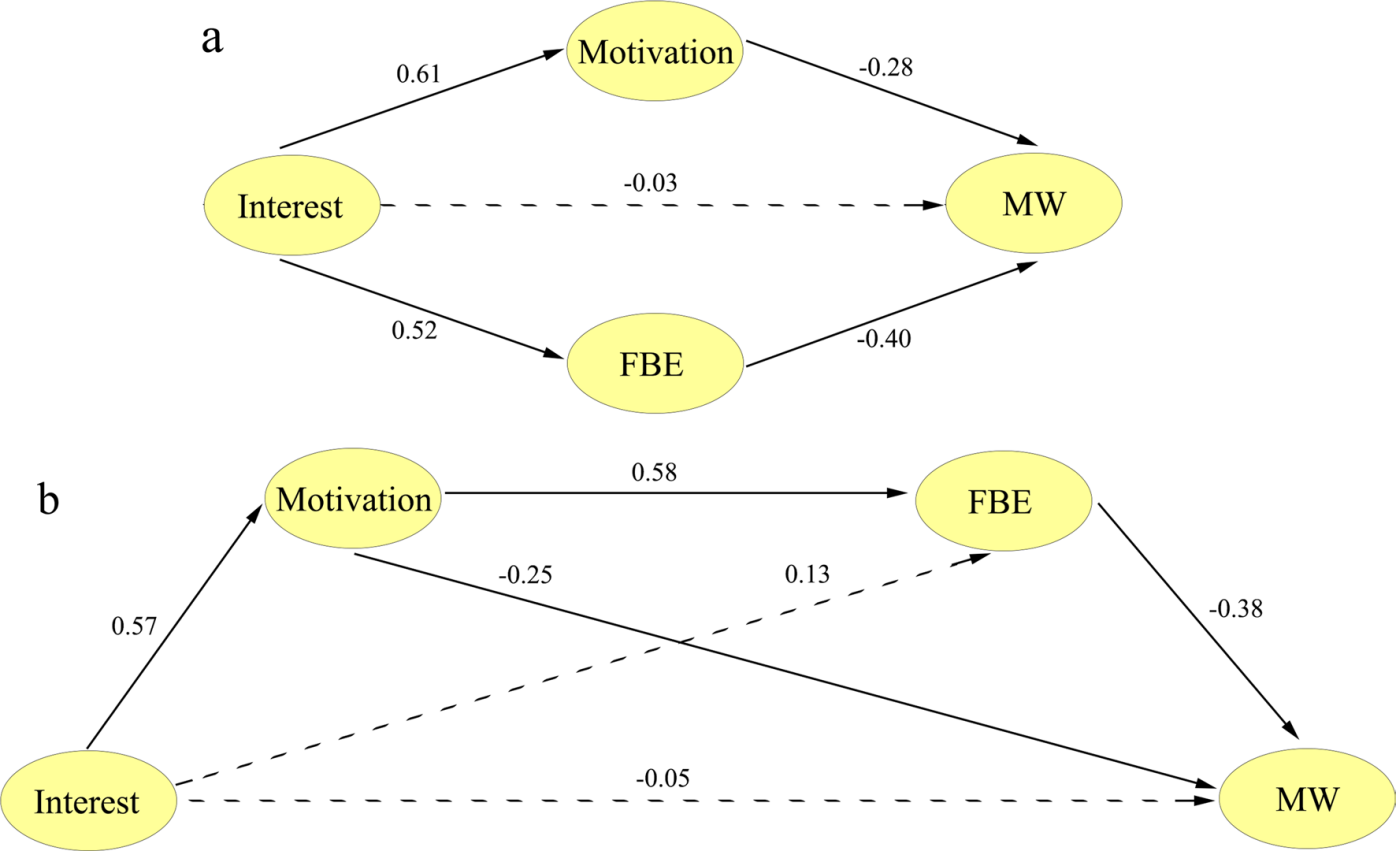
**a** The parallel mediation model examining how motivation and focus back effort jointly mediated the relationship between interest and mind wandering. **b** The structural equation model investigating the sequential mediation of motivation and focus back effort in the association between interest and mind wandering. The contribution of each latent variable to the other is represented by a single-headed arrow from one latent variable (circle) to another. Solid paths indicate significant relationships at *p* < 0.05, while dotted lines indicate non-significant relationships at *p* < 0.05. To simplify the interpretation, the factor loadings of manifest variables have been excluded from the illustration and can be found in Additional file 1: Table S7

SEM analyses were performed to examine the first research question, which aimed to explore the impact of focus back effort, motivation, and interest on mind wandering. Building upon the results of the CFA and the findings by Unsworth et al. (2013) that the motivation latent variable mediates the relationship between interest and mind wandering latent variables, as well as the previous discovery that focus back effort can be influenced by the domain-specific variable (He et al., 2021a, 2021b), two models were examined. Model 1, represented in Fig. 2a, proposed a parallel mediation model where both motivation and focus back effort mediated the association between interest and mind wandering. Model 1 showed: *χ*^2^ (49) = 145.75, RMSEA = 0.10, SRMR = 0.10, NNFI = 0.91, CFI = 0.94, AIC = 203.75. Conversely, Fig. 2b presented Model 2, which illustrated the SEM with motivation and focus back effort sequentially mediating the relationship between interest and mind wandering. Model 2 demonstrated a better fit to the data, *χ*^2^ (48) = 103.38, RMSEA = 0.07, SRMR = 0.04, NNFI = 0.95, CFI = 0.96, AIC = 163.38. AIC, a measure considering model complexity relative to the number of parameters (Akaike, 1973), favors models with lower values. Notably, the AIC of Model 2 was lower than that of Model 1, indicating the superiority of Model 2. The results of the chain mediation analysis revealed that the direct path from interest to mind wandering was not significant (*β* = − 0.050, *p* = 0.579), whereas motivation and focus back effort sequentially mediated this relationship (indirect effect = − 0.317, *p* = 0.002). The mediation process involved three indirect effects: path 1 (interest → motivation → mind wandering; indirect effect = − 0.141), path 2 (interest → focus back effort → mind wandering; indirect effect = − 0.051), and path 3 (interest → motivation → focus back effort → mind wandering; indirect effect = − 0.125). The bootstrap 95% CI of path 2 contained 0 (− 0.137, 0.016), suggesting that path 2 was not statistically significant. However, the bootstrap 95% CI of path 1 and path 3 did not contain 0 (path 1: − 0.291, − 0.028; path 2: − 0.237, − 0.054), suggesting significant indirect effects for path 1 and path 3. Notably, path 1 and path 3 remained significant after family-wise SGoF multiple comparison correction. These findings suggest that focus back effort, in addition to the established mediating role of motivation in the relationship between interest and mind wandering (Unsworth & McMillan, 2013), plays a significant mediating role.

Considering that the motivation and interest questions were administered after the focus back effort and mind wandering questions in each task, we constructed an additional model (Additional file 2: Fig. S1) to explore the prediction of motivation and interest by focus back effort and mind wandering. However, the path linking focus back effort to mind wandering to interest and, finally, to motivation was found to be nonsignificant.

#### Mediation models including task performance

**Fig. 3:**
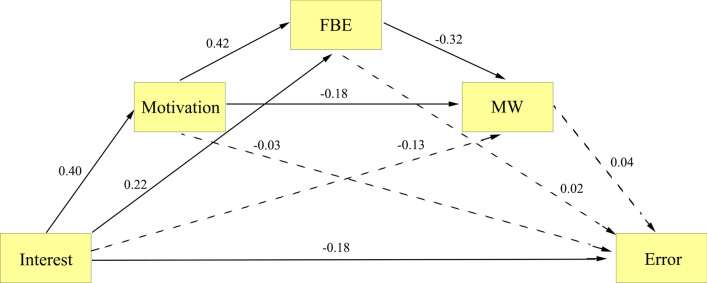
Mediation effect paths of motivation, focus back effort (FBE), and mind wandering (MW) between interest and error in the sustained attention to response task (Model 3). The single-headed arrow from one (square) to another variable represents the effect of that variable on the other variable. All numbers appearing beside each arrow are standardized. All solid paths indicate significance at *p* < 0.05, while dotted lines indicate non-significant effects at *p* < 0.05

The correlation table (Additional file 1: Table S5) indicates a near-zero correlation between interest and error in the 0-back. Consequently, we focused on task error in the SART (Model 3) and the 1-back task (Model 4) to address the second research question. The results of Model 3 indicated direct influence of interest on task performance (direct effect = − 0.178, *p* = 0.016, as shown in Fig. 3). Furthermore, none of the other variables mediated the relationship between interest and task performance (indirect effect = − 0.017, *p* = 0.702).

**Fig. 4:**
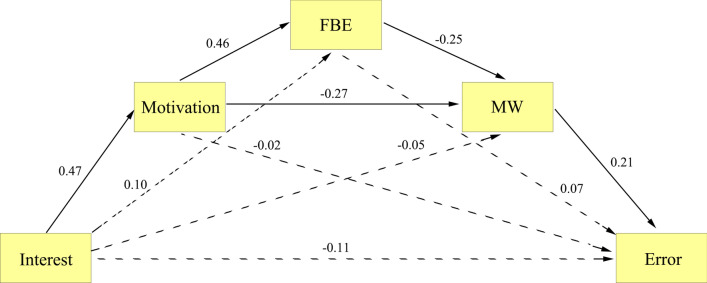
Mediation effect paths of motivation, focus back effort (FBE), and mind wandering (MW) between interest and error in the 1-back task (Model 4). The single-headed arrow from one (square) to another variable represents the effect of that variable on the other variable. All numbers appearing beside each arrow are standardized. All solid paths are significant at *p* < 0.05 whereas all dotted lines are not significant at *p* < 0.05

The results of Model 4, utilizing variables from the 1-back task, indicated that interest did not directly predict error (direct effect = − 0.108, *p* = 0.177, see Fig. 4). The mediation analysis showed seven indirect effects: path 1 (interest → motivation → error; indirect effect = − 0.010), path 2 (interest → motivation → focus back effort → error; indirect effect = 0.015), path 3 (interest → motivation → mind wandering → error; indirect effect = − 0.055), path 4 (interest → motivation → focus back effort → mind wandering → error; indirect effect = − 0.011), path 5 (interest → focus back effort → error; indirect effect = 0.007), path 6 (interest → focus back effort → mind wandering → error; indirect effect = − 0.005), and path 7 (interest → mind wandering → error; indirect effect = − 0.011). The bootstrap 95% CI of paths 3 and 4 did not include 0 (path 3: − 0.061, − 0.006; path 4: − 0.030, − 0.002), indicating significant indirect effects. However, the bootstrap 95% CIs of the other paths included 0 (path 1: − 0.090, 0.086; path 2: − 0.017, 0.056; path 5: − 0.005, 0.039; path 6: − 0.025, 0.0005; path 7: − 0.060, 0.014), suggesting non-significant indirect effects. Path 3 and path 4 remained significant even after applying the SGoF multiple comparison correction. The significant indirect of path 3 is consistent with the findings of Unsworth et al. (2013). The significant indirect effect of path 4 supports the resource-control hypothesis, indicating that individuals adjust executive control based on motivational factors to allocate resources between mind wandering and the current task, which impacts performance outcomes (Thomson et al., 2015a). The reason why path 3 and path 4 were only significant in the 1-back task (but not the SART and 0-back task) may be due to the fact that mind wandering has a greater detrimental effect on tasks under more demanding conditions (Greve & Was, 2022; Rummel & Boywitt, 2014).

## Study 2

Overall, the results of Study 1 are consistent with our hypotheses. In Study 2, a reading comprehension task was employed to validate the findings obtained in Study 1. This task was chosen due to its moderate to high difficulty, high ecological validity, frequent use in mind wandering research, and assessing the individual’s comprehension score of learned material is a common approach to measuring competence and ability in diverse settings, particularly in the field of education (Mcvay & Kane, 2012b; Soemer & Schiefele, 2019, 2020; Unsworth & McMillan, 2013). Additionally, previous research has demonstrated a significant correlation between reading comprehension and individual differences in motivation and interest (Robison & Unsworth, 2015; Unsworth & McMillan, 2013). Mediation models were employed to analyze the data in Study 2.

### Methods

#### Participants

In the second study, a total of 144 participants aged 18–38 (mean age = 21.61, *SD* = 2.84; 95 females, 49 males) from universities with normal or corrected-to-normal vision were recruited. Participants provided informed consent in accordance with the Declaration of Helsinki before completing a reading comprehension task. The study protocol was approved by the Ethics Committee in China. The sample size was determined based on the standardized coefficients obtained from the 1-back mediation model in Study 1. To calculate the necessary sample size, power analysis for the mediation model was conducted using an R application (R Core Team, 2016) (Schoemann et al., 2017). The target power was set at 0.8, and the confidence level for all indirect effects was set at 95%. The analysis indicated that a total sample size of 144 participants would be required to achieve 80% power in detecting the effect.

#### Procedure

Participants completed the reading comprehension task in a well-lit computer classroom after providing their consent forms.

#### Apparatus

The apparatus utilized in this study was identical to that employed in Study 1.

#### Reading comprehension task

The test materials employed in the second study consisted of an article extracted from Volume 5 of Morley’s “Chinese Reading Level Test (Revised Edition)” along with 9 multiple-choice reading comprehension questions (Kong et al., 2018). This article is a descriptive essay titled “I Gave My Whole Life to Him.” and consisted 1297 Chinese characters and was presented in a page-by-page format, with each page containing approximately 150 to 250 Chinese characters. The text was displayed using a dark gray serif font on a white background. To simulate natural reading, participants were provided with the flexibility to navigate through the pages using the left and right arrow buttons on a keyboard. Participants were given a time limit of 6 min to complete the reading of the article. Subsequently, participants were instructed to answer a set of 9-item multiple-choice questions based on their comprehension of the text. No specific time restriction was imposed for answering the questions, allowing participants as much time as needed. Each correct response was awarded 1 point while each incorrect response was scored 0 points. The test is relatively simple, and in previous studies, the majority of participants were able to achieve above-average scores (Kong et al., 2018).

*Thought probes* Six probes were utilized to measure both mind wandering and focus back effort during the reading task. Each thought probe was randomly administered to participants within a time window ranging from 30 s to 1.5 min.

*Interest and motivation* After reading the text and completing the reading comprehension tests, participants were requested to respond to two inquiries regarding their general level of interest in the text and their motivation to perform well on the test.

Participants completed the reading comprehension test and rated the thought probes utilizing the computer mouse.

#### Statistical analysis

The HLM approach was employed to examine the potential decrease in focus back effort over time, as detailed in the Additional file 1. The calculation of the mind wandering score and focus back effort score followed the same methodology as outlined in Study 1. The reading comprehension score was derived by summing the individual score for each question. We employed the same methods as Study 1 for conducting correlational analysis, mediation analysis, and multiple comparison correction.

### Results and discussion

#### Descriptive statistics

In Additional file 1: Table S8 presents the descriptive statistics for the variables in the present study. The comprehension tests showed good Cronbach’s alpha reliability coefficients (*α* = 0.803). When the participants reported being completely on task, the selection for the second question indicated that they made no attempt to refocus their attention on the task. The outcomes showed that all variables exhibited a normal distribution. The Pearson correlation coefficients for the measures used in Study 2 are presented in Additional file 1: Table S9. The HLM analysis revealed a decrease in focus back effort throughout the duration of the reading comprehension task, as shown in Additional file 1: Table S10. The findings indicated that some individuals reported high focus back effort but low motivation and some individuals demonstrated low focus back effort but high motivation (Additional file 1: Table S6).

#### Mediation model including task performance

**Fig. 5:**
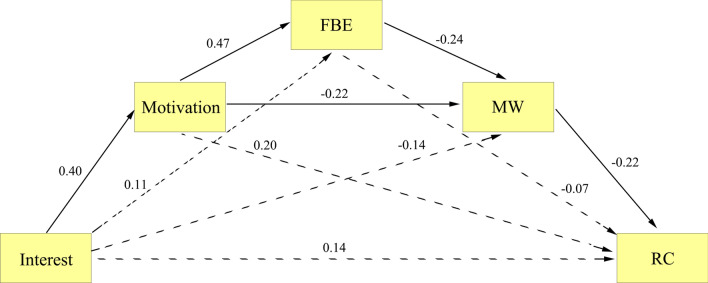
Mediation effect paths of motivation, focus back effort (FBE), and mind wandering (MW) between interest and reading comprehension (RC) in Study 2 (Model 5). The single-headed arrow from one (square) to another variable represents the effect of that variable on the other variable. All numbers appearing beside each arrow are standardized. All solid paths are significant at *p* < 0.05 whereas all dotted lines are not significant at *p* < 0.05

We then aimed to validate the mediation effects obtained in Study 1. Model 5, utilizing variables from the reading comprehension task, yielded a non-significant direct path from interest to reading comprehension (direct effect = 0.139, *p* = 0.89; refer to Fig. 5). The mediation analysis identified seven indirect effects: path 1 (interest → motivation → reading comprehension; indirect effect = 0.080), path 2 (interest → motivation → focus back effort → reading comprehension; indirect effect = − 0.033), path 3 (interest → motivation → mind wandering → reading comprehension; indirect effect = 0.019), path 4 (interest → motivation → focus back effort → mind wandering → reading comprehension; indirect effect = 0.010), path 5 (interest → focus back effort → reading comprehension; indirect effect = − 0.019), path 6 (interest → focus back effort → mind wandering → reading comprehension; indirect effect = 0.006), and path 7 (interest → mind wandering → reading comprehension; indirect effect = 0.030). The bootstrap 95% CIs for paths 3 and 4 did not contain 0 (path 3: 0.002, 0.057; path 4: 0.002, 0.032), indicating significant indirect effects. However, the bootstrap 95% CIs for the other paths did contain 0 (path 1: − 0.011, 0.192; path 2: − 0.078, 0.001; path 5: − 0.067, 0.004; path 6: − 0.001, 0.027; path 7: − 0.004, 0.103), indicating no significant indirect effects. Consistent with the findings of Study 1, Path 3 and path 4 remained significant after applying family-wise SGoF multiple comparison correction in this study. The results of Study 1 and Study 2 indicate consistent cross-task mediating effects.

As anticipated, the path from focus back effort to mind wandering to interest and then to motivation was found to be statistically non-significant (Additional file 3: Fig. S2), aligning with the results obtained in Study 1.

## General discussion

The present studies examined the role of focus back effort in the relationships among interest, motivation, and mind wandering. Two studies were conducted to address our research questions. The findings revealed that focus back effort exhibited a decline over time and was subject to modulation by individuals based on task demands. In Study 1, CFA results demonstrated significant correlations among interest, motivation, focus back effort, and mind wandering. Furthermore, we examined whether focus back effort mediates the relationship between individual differences in mind wandering, motivation, and interest. As expected, the results showed a significant indirect effect of interest → motivation → focus back effort → mind wandering. The mediation model based on the 1-back task revealed that high interest facilitated task performance through increased motivation, focus back effort, and decreased mind wandering. These findings were consistent with the results obtained in the reading comprehension task of Study 2 where the indirect effect remained significant.

One of the fundamental principles of the resource-control theory is the decline of executive control as a task progresses (Thomson et al., 2015a). The HLMs conducted in both studies of this work consistently demonstrated that focus back effort faded over time, which is consistent with the predictions of the resource-control hypothesis. The resource-control theory suggests that there exists a limited pool of cognitive resources that are shared between task-related thoughts and mind wandering, and the allocation of these resources is context-dependent, such as the task demand (Thomson et al., 2015a). The findings of Study 1 further support the resource-control theory by revealing higher levels of focus back effort during high-load tasks compared to low-load tasks. Given that focus back effort has been shown to be correlated with mind wandering in both the current studies and previous research (He et al., 2021a, 2021b) and that linked to functional connectivity between regions of the executive network (He et al., 2021a, 2021b), the findings of the present studies provide support to the notion that focus back effort may reflect the adjustment of executive control in allocating resources between mind wandering and the task at hand.

The results of the CFA revealed significant associations among focus back effort, motivation, and interest. These results support the demonstration that motivation assumes the role of adjustment of resources (Pessoa, 2009) and align with the resource-control theory of mind wandering, which suggests that motivation facilitates the adjustment of executive control, as reflected by focus back effort, towards task-relevant stimuli (Thomson et al., 2015a). Importantly, the positive relationship between concentration and motivation and interest (Kane et al., 2007; Mittner et al., 2016) is in line with the interpretation of focus back effort as a form of concentration in a prior study (He et al., 2021a, 2021b). Furthermore, the results suggest that individual differences in motivation and interest play important roles in mind wandering. Specifically, participants who reported more episodes of mind wandering also reported lower levels of motivation to perform well and less interest in the task compared to those who reported fewer mind wandering episodes. These findings are consistent with previous research (Antrobus et al., 1966; Grodsky & Giambra, 1990; Jackson & Balota, 2012; Unsworth & McMillan, 2013).

The key finding of this study is that interest predicts motivation, and focus back effort mediates the relationship between motivation and mind wandering. The results highlight the significant influence of both motivation and the sequential mediation of motivation and focus back effort on the association between interest and mind wandering. This indicates that the impact of interest on mind wandering is contingent upon the levels of both motivation and focus back effort. Motivational factors and interest have been suggested to be highly correlated (Hidi & Harackiewicz, 2000; Robison et al., 2020), indicating that the more interested individuals are in a task, the more motivated they are to perform well. Noncognitive variables such as motivation and interest are typically investigated to examine the influence of specific psychological states on mind wandering (e.g., Jordano & Touron, 2017; Robison & Unsworth, 2015; Unsworth & McMillan 2013, 2017). Our results expand on the findings of Unsworth et al. (2013) by adding focus back effort to the model and demonstrating that individual differences in focus back effort play an important role in this relationship. It should be noted that interest indirectly influenced mind wandering through two mediation pathways. Individuals who are more interested in performing a task may be more motivated to do the work, consequently leading to fewer occurrences of mind wandering. This can be attributed, in part, to their enhanced allocation of executive control, as reflected by focus back effort, to redirect their attention back to the task at hand when their thoughts drift away (Thomson et al., 2015a). The resource-control theory posits that executive control serves not only to inhibit mind wandering but also to sustain goal maintenance. However, focus back effort in the current study primarily focuses on inhibiting mind wandering and does not directly measure the maintenance of the target task. Thus, the applicability of the current study’s results to the broader aspects of the resource-control theory should be approached with caution.

In terms of individual differences in task performance, the present studies conducted several mediation analyses to explore the underlying mechanisms. The results of these analyses align with previous research (Unsworth & McMillan, 2013) and support the indirect effect of interest on task performance. Specifically, consistent with the attentional resource hypothesis (Smallwood & Schooler, 2006) and the resource-control theory (Thomson et al., 2015a), the model demonstrated validity only for the 1-back task and the reading comprehension task. The findings revealed that interest influenced task performance through two distinct pathways. These pathways suggest that participants with high motivation may exhibit better task performance due to their frequent refocusing when in a mind wandering state, which leads to reduced engagement in mind wandering. Higher levels of interest, motivation, and focus back effort were associated with improved task performance by reducing mind wandering, which is detrimental to performance when available cognitive resources are insufficient to accommodate mind wandering. These findings are consistent with the previous study by Greve and Was (2022) and Rummel and Boywitt (2014), indicating a demand-sensitive relationship between mind wandering and task performance. Moreover, these findings align with the resource-control theory (Thomson et al., 2015a), which proposes that the negative effect of mind wandering on task performance does not always occur to if certain tasks do not require complete allocation of attentional resources, allowing for instances where mind wandering can occur without significant performance costs.

While previous research (He et al., 2021a, 2021b) did not find a significant relationship between mind wandering and focus back effort during daily life, the present studies, along with another study by He et al., (2021a, 2021b), established a clear negative relationship between mind wandering and focus back effort in laboratory settings. It is suggested that this disparity may be attributed to the presence of other distractions in daily life that mask the relationship between mind wandering and focus back effort. In everyday situations, individuals may attempt to refocus their attention on the task at hand, but these efforts might be impeded by various distractions that make it challenging for this process to successfully draw attention back to the task.

In addition to the resource-control theory, the present study may also have implications for other theories unrelated to executive control. As noted by Wong et al. (2022) in a recent review of the switching perspective of mind wandering, motivation may induce a biased metacontrol toward task persistence (Wong et al., 2022). Mental set switching or shifting allows us to adjust our thoughts based on changing priorities (Diamond, 2013; Miyake et al., 2000), and this process requires inhibitory control (Diamond, 2013). With regard to mind wandering, Wong et al. (2022) proposed that increased frequency of mind wandering is associated with a metacontrol bias toward cognitive flexibility (i.e., weak inhibition of both mind wandering states and task-related thoughts). According to this perspective, participants should allocate more attentional resources to the current task and reduce the resources devoted to task-unrelated thoughts due to the metacontrol setting biased toward persistence. In this regard, focus back effort may also indicate a metacontrol, and this assumption requires further investigation in future research.

According to the process-occurrence framework, mind wandering can be conceptualized as consisting of separate onset and maintenance processes (Smallwood, 2013). Executive control plays a role in both directly inhibiting the initiation of mind wandering and indirectly influencing its persistence. The direct effect is manifested by inhibiting the initiation of mind wandering, while the indirect effect is manifested by maintaining the continuation of mind wandering. As focus back effort has been demonstrated to be a process that occupies cognitive resources (He et al., 2021a, 2021b), the results of the current studies might extend the process-occurrence framework and provide support for the notion that executive control resources could also be utilized to inhibit the continuation of mind wandering. However, unlike the hypotheses introduced in the Introduction section, this framework specifically addresses the dynamic process of mind wandering, which was not explored in the current study paradigm. Further research is needed to investigate this issue. Eye-tracking may offer a valuable tool for obtaining more direct evidence pertaining to the process of focus-back effort and mind wandering.

Regarding the effort involved in redirecting attention back to the current activity while experiencing mind wandering, our previous and current studies were the pioneering investigations of this construct, and we coined the term “focus back effort”. It is crucial to emphasize that focusing back on one’s task was supposed to require effort in the present studies (He et al., 2021a, 2021b; He et al., 2021a, 2021b). However, it is plausible that there are instances where refocusing occurs effortlessly. There could be a habituation mechanism that automatically ends mind wandering after a specific period, leading us to shift our attention back to the task without any conscious effort. Hence, it would be valuable to investigate the characteristics of effortless focus back in future research. In addition, although the present studies provide support for the idea that focus back effort may partially reflect the regulation of executive control, which is proposed by the resource-control theory, it is important to acknowledge that this component is likely to be more intricate and necessitates further exploration in future investigations. Furthermore, future research should continue to seek evidence to establish its distinctiveness from executive control and mind wandering.

Although our results provide valuable insights into mind wandering, it is important to acknowledge and address several limitations. First, our study focused exclusively on the variation explained by motivation, interest, and focus back effort in predicting mind wandering. However, other factors such as working memory capacity (Unsworth & McMillan, 2013), mood (Smallwood et al., 2009a, 2009b), and personal concerns (Smallwood & Schooler, 2006) may also be important in explaining the variance in mind wandering. Future research should aim to explore the contribution of these additional factors in understanding mind wandering. Second, our sample consisted solely of college students, and it is well-established that age is a crucial factor that influences individual differences in mind wandering (Jackson & Balota, 2012). Therefore, caution must be exercised when generalizing our findings to a broader population. Third, our studies primarily focused on examining the relationships among individual differences in motivation, interest, focus back effort, mind wandering, and task performance. However, it is important to recognize that mind wandering encompasses various types, including mind wandering with and without awareness, as well as intentional and unintentional mind wandering (Seli et al., 2016a, 2016b, 2016c). Moreover, the causal evidence for the involvement of these factors in mind wandering is limited. Future research should aim to investigate the influence of these factors on different types of mind wandering and establish causal relationships. Furthermore, it is recommended to explore and differentiate other potential responses, such as task-related interference and external distraction (Stawarczyk et al., 2011). Further investigation into these dimensions is warranted to enhance our understanding of the complex nature of mind wandering. In this study, we examined the relationships between variables from an individual difference perspective. However, our definition of focus back effort implies that it occurs after mind wandering. Therefore, the process of transitioning from mind wandering to focus back effort deserves further investigation. Additionally, considering that both mind wandering and focus back effort are sensitive to time on task, it is worth exploring whether the importance of focus back effort changes as individuals spend more time on a task.

This article contributes to our understanding of the factors influencing mind wandering. Adopting an individual difference perspective, these studies demonstrate the predictive role of interest in mind wandering, mediated by motivation and focus back effort. Given that mind wandering occurs across various activities, including creative thinking (Baird et al., 2012), driving (Yanko & Spalek, 2014), academic achievement (Mrazek et al., 2017), and workplace performance (Sridar & Kennedy, 2012), these findings have important implications for further research in these domains. Moreover, the present studies offer potential insights for the development of interventions targeting mind wandering, which can significantly impact students' classroom engagement and comprehension performance (Franklin et al., 2011; Mrazek et al., 2017; Seli et al., 2016a, 2016b, 2016c). The results of the current work provide support for this perspective, particularly Study 2, which revealed that focus back effort mediated the relationship between motivation and mind wandering in a reading comprehension task (although not the only mediating pathway). Previous research has extensively explored the connection between motivation and mind wandering (Klinger, 2008; Seli et al., 2015; 2016a, 2016b, 2016c; Unsworth & McMillan, 2013), and examined the effects of manipulating individuals' motivation on mind wandering (Seli et al., 2017a, 2017b). Consequently, focus back effort emerges as a potentially important target for interventions aimed at mitigating mind wandering. Thus, in educational contexts, interventions focused on enhancing focus back effort may lead to improved academic performance. Similarly, in work settings, relevant research may contribute to reducing the detrimental impact of mind wandering on job performance. By addressing the role of focus back effort, these interventions have the potential to provide practical solutions to minimize the negative consequences of mind wandering in various domains.

## Conclusions

In summary, this study builds upon previous research examining the interplay between mind wandering, motivation, interest, and task performance by introducing focus back effort as a novel factor. The study demonstrates that interest indirectly predicts mind wandering through the mediating pathways of motivation and focus back effort. Furthermore, interest indirectly influences task performance by impacting motivation, focus back effort, and mind wandering in high-load tasks. These results shed light on the underlying mechanism of focus back effort in the relationships among motivation, interest, mind wandering, and task performance, underscoring the pivotal role of executive control adjustments in resource allocation when considering domain-specific factors and mind wandering. In general, the results align with the resource-control theory of mind wandering (Thomson et al., 2015a) and offer practical implications for deepening our understanding of the factors influencing mind wandering.

## Supplementary Information

**Additional file 1**. Supplementary methods and results.

**Additional file 2**. Mediation effect paths of mind wandering and interest between focus back effort and motivation in Study 1.

**Additional file 3**. Mediation effect paths of mind wandering , interest, and motivation between focus back effort and reading comprehension in Study 2.

## Data Availability

The datasets generated and/or analyzed during the current studies are available in the figshare repository, https://doi.org/10.6084/m9.figshare.21294963. These studies were not preregistered.

## Abbreviations

SART: Sustained attention to response task
CFA: Confirmatory factor analysis
MW: Mind wandering
FBE: Focus back effort
SEM: Structural equation model
HLM: Hierarchical linear modeling
χ^2^: Chi-square statistics
RMSEA: Root mean square error of approximation
SRMR: Standardized root mean residual
NNFI: Non-normed fit index
CFI: Comparative fit index
CI: Confidence interval
SGoF: Sequential goodness of fit
MW-SART: Mind wandering in the sustained attention to response task
MW-0B: Mind wandering in the 0-back task
MW-1B: Mind wandering in the 1-back task
FBE-SART: Focus back effort in the sustained attention to response task
FBE-0B: Focus back effort in the 0-back task
FBE-1B: Focus back effort in the 1-back task
Motivation-SART: Motivation in the sustained attention to response task
Motivation-0B: Motivation in the 0-back task
Motivation-1B: Motivation in the 1-back task
Interest-SART: Interest in the sustained attention to response task
Interest-0B: Interest in the 0-back task
Interest-1B: Interest in the 1-back task
