# Supplementary material for: The role of focus back effort in the relationships among motivation, interest, and mind wandering: an individual difference perspective
Source: Cogn Res Princ Implic. 2023 Jul 13;8:43. doi: 10.1186/s41235-023-00502-0 (PMC10344852; doi:10.1186/s41235-023-00502-0)
Supplement: Supplementary file 1 — Additional file 1. Supplementary methods and results. [file 41235_2023_502_MOESM1_ESM.docx]

**Supplementary Methods**

Hierarchical linear models (HLMs) were utilized to explore data with trials nested within participants regarding the influence of time on task on focus back effort. The data was analyzed using the lmerTest package in R (Kuznetsova et al., 2017). In Study 1, a total of 262 trials were completed in the SART, while 275 trials were completed in both the 0-back and 1-back tasks. Thought probes were randomly presented before 10 of those trials in the SART and at the end of each block in the N-back tasks. In Study 2, trial numbers were coded as 1, 2, 3, 4, 5, and 6. Consistent with previous research (Marcusson-Clavertz et al., 2019), trial numbers were restricted to the trials that preceded the probes and standardized by dividing the within-person centered scores by the within-person SDs. Data points in which participants answered between 2 and 6 for the first mind wandering question were included in Models S1 (SART), S2 (0-back task), S3 (1-back task), and S4 (reading comprehension task). Therefore, Model S1 included 1675 data points, Model S2 included 1754 data points, Model S3 included 1608 data points, and Model S4 included 717 data points.

**Supplementary Results**

The results indicated that the kurtosis and skewness values for all measures in both studies were within acceptable ranges (skewness < 2; kurtosis < 4), except for task performance measures in Study 1 (Supplementary Tables A and G), which deviated from a normal distribution (Kline, 1998). Specifically, the skewness and kurtosis values of the three tasks' errors in Study 1 were outside the acceptable ranges, indicating non-normal distributions. To address this issue, a square root transformation (Seli et al., 2016) was applied to these data to achieve acceptable levels of kurtosis and skewness (omission error of SART: transformed skewness = -0.141 and transformed kurtosis = 0.582; 0-back error: transformed skewness = 0.367 and transformed kurtosis = 0.579; 1-back error: transformed skewness = 0.660 and transformed kurtosis = 1.674). Furthermore, the results demonstrated that focus back effort exhibited a decrease over time within each task (refer to Tables S2, S3, and S4).

The results of correlation analyses in Study 1 (see Table S5) suggested significant relationships between mind wandering, focus back effort, motivation, and interest in each task. Specifically, significant correlations were observed between mind wandering and focus back effort (*rs* < -0.406, *ps* < 0.001), focus back effort and motivation (*rs* > 0.447, *ps* < 0.001), focus back effort and interest (*rs* > 0.263, *ps* < 0.001), and motivation and interest (*rs* > 0.377, *ps* < 0.001). Furthermore, significant relationships were found between mind wandering and motivation (*rs* < -0.393, *ps* < 0.001), and between mind wandering and interest (*rs* < -0.276, *ps* < 0.001). The correlation results revealed that the significant relationship between mind wandering and error was only present in the 1-back task (*r* = 0.217, *p* = 0.002), while there was no significant correlation between omission error and mind wandering in the SART (*r* = 0.031, *p* = 0.662) or between error and mind wandering in the 0-back task (*r* = 0.032, *p* = 0.650). The results are consistent with the attentional resources hypothesis (Smallwood & Schooler, 2006) and the resource-control theory (Thomson et al., 2015), which suggest that available cognitive resources are essential to control attention and maintain focus. The correlation results in Study 2 indicated significant relationships between mind wandering, focus back effort, motivation, and interest (Table S8). Moreover, reading comprehension was significantly correlated with mind wandering, motivation, and interest (*ps* < 0.01).

**Table S1** Descriptive statistics for variables in Study 1.

| Measure | *M* | *SD* | Range | Skewness | Kurtosis |
| --- | --- | --- | --- | --- | --- |
| MW-SART | 2.98 | 1.05 | 1.10-6 | 0.48 | -0.32 |
| MW-0B | 3.19 | 1.10 | 1.40-6 | 0.51 | -0.38 |
| MW-1B | 2.81 | 1.00 | 1.10-6 | 0.45 | -0.18 |
| FBE-SART | 3.67 | 1.03 | 1-6 | -0.22 | -0.18 |
| FBE-0B | 3.44 | 1.25 | 1-6 | -0.18 | -0.82 |
| FBE-1B | 3.87 | 1.08 | 1-6 | -0.45 | -0.20 |
| Motivation-SART | 3.92 | 1.41 | 1-6 | -0.37 | -0.65 |
| Motivation-0B | 3.95 | 1.31 | 1-6 | -0.59 | -0.37 |
| Motivation-1B | 4.13 | 1.33 | 1-6 | -0.43 | -0.53 |
| Interest-SART | 2.54 | 1.19 | 1-6 | 0.55 | -0.25 |
| Interest-0B | 2.29 | 1.24 | 1-6 | 0.71 | -0.27 |
| Interest-1B | 2.72 | 1.33 | 1-6 | 0.39 | -0.68 |
| Omission error of SART | 0.04 | 0.06 | 0-0.28 | 2.54 | 6.52 |
| Error-0B | 0.05 | 0.05 | 0-0.36 | 2.62 | 10.11 |
| Error-1B | 0.11 | 0.12 | 0-0.80 | 2.93 | 11.63 |

Note. MW-SART = mind wandering in the sustained attention to response task; MW-0B = mind wandering in the 0-back task; MW-1B = mind wandering in the 1-back task; FBE-SART = focus back effort in the sustained attention to response task; FBE-0B = focus back effort in the 0-back task; FBE-1B = focus back effort in the 1-back task; Motivation-SART = motivation in the sustained attention to response task; Motivation-0B = motivation in the 0-back task; Motivation-1B = motivation in the 1-back task; Interest-SART = interest in the sustained attention to response task; Interest-0B = interest in the 0-back task; Interest-1B = interest in the 1-back task.

**Table S2** Fixed effects of the multilevel logistic regression model (Model S1): focus back effort in the sustained attention to response task.

|  | Estimate | *SE* | *z* | *p* | Odds ratio | 95% CI |
| --- | --- | --- | --- | --- | --- | --- |
| (Intercept) | 3.72 | 0.07 | 54.16 | < 0.001 | 41.39 | (36.17, 47.42) |
| Trial | -0.05 | 0.01 | -6.44 | < 0.001 | 0.95 | (0.94, 0.97) |

Note. Confidence intervals of 95% are for the odds ratio. This model was based on 1675 observations.

**Table S3** Fixed effects of the multilevel logistic regression model (Model S2): focus back effort in the 0-back task.

|  | Estimate | *SE* | *z* | *p* | Odds ratio | 95% CI |
| --- | --- | --- | --- | --- | --- | --- |
| (Intercept) | 3.73 | 0.07 | 50.04 | < 0.001 | 41.85 | (36.14, 48.48) |
| Trial | -0.04 | 0.01 | -5.40 | < 0.001 | 0.96 | (0.95, 0.96) |

Note. Confidence intervals of 95% are for the odds ratio. This model was based on 1754 observations.

**Table S4** Fixed effects of the multilevel logistic regression model (Model S3): focus back effort in the 1-back task.

|  | Estimate | *SE* | *z* | *p* | Odds ratio | 95% CI |
| --- | --- | --- | --- | --- | --- | --- |
| (Intercept) | 3.96 | 0.07 | 57.38 | < 0.001 | 52.20 | (45.58, 59.80) |
| Trial | -0.06 | 0.01 | -7.52 | < 0.001 | 0.94 | (0.93, 0.96) |

Note. Confidence intervals of 95% are for the odds ratio. This model was based on 1608 observations.

**Table S5** Correlation coefficients for all measures in Study 1.

| Measure | 2 | 3 | 4 | 5 | 6 | 7 | 8 | 9 | 10 | 11 | 12 | 13 | 14 | 15. Error-1B |
| --- | --- | --- | --- | --- | --- | --- | --- | --- | --- | --- | --- | --- | --- | --- |
| 1. MW-SART | 0.78** | 0.75** | -0.46** | -0.47** | -0.43** | -0.39** | -0.39** | -0.41** | -0.33* | -0.24** | -0.23** | 0.10 | 0.05 | 0.06 |
| 2. MW-0B |  | 0.68** | -0.39** | -0.44** | -0.39** | -0.40** | -0.44** | -0.41** | -0.27** | -0.28** | -0.26** | 0.21 | 0.03 | 0.09 |
| 3. MW-1B |  |  | -0.29** | -0.35** | -0.41** | -0.33** | -0.28** | -0.42** | -0.26** | -0.19** | -0.26** | 0.15* | 0.10 | 0.22** |
| 4. FBE-SART |  |  |  | 0.69** | 0.72** | 0.51** | 0.47** | 0.48** | 0.39** | 0.34** | 0.26** | -0.08 | 0.05 | 0.01 |
| 5. FBE-0B |  |  |  |  | 0.68** | 0.43** | 0.45** | 0.42** | 0.37** | 0.26** | 0.24** | -0.17* | -0.05 | -0.02 |
| 6. FBE-1B |  |  |  |  |  | 0.46** | 0.38** | 0.50** | 0.34** | 0.29** | 0.32** | -0.16* | 0.01 | -0.10 |
| 7. Motivation-SART | |  |  |  |  |  | 0.71** | 0.74** | 0.40** | 0.30** | 0.41** | -0.11 | 0.04 | -0.08 |
| 8. Motivation-0B | |  |  |  |  |  |  | 0.61** | 0.38** | 0.38** | 0.38** | -0.07 | 0.03 | -0.02 |
| 9. Motivation-1B | |  |  |  |  |  |  |  | 0.38** | 0.33** | 0.47** | -0.06 | -0.01 | -0.13 |
| 10. Interest-SART | |  |  |  |  |  |  |  |  | 0.62** | 0.65** | -0.20** | 0.04 | -0.02 |
| 11. Interest-0B |  |  |  |  |  |  |  |  |  |  | 0.66** | -0.08 | 0.05 | -0.12 |
| 12. Interest-1B |  |  |  |  |  |  |  |  |  |  |  | -0.14* | -0.03 | -0.15* |
| 13. Omission error of SART | | |  |  |  |  |  |  |  |  |  |  | 0.22** | 0.25** |
| 14. Error-0B |  |  |  |  |  |  |  |  |  |  |  |  |  | 0.31** |

Note. MW-SART = mind wandering in the sustained attention to response task; MW-0B = mind wandering in the 0-back task; MW-1B = mind wandering in the 1-back task; FBE-SART = focus back effort in the sustained attention to response task; FBE-0B = focus back effort in the 0-back task; FBE-1B = focus back effort in the 1-back task; Motivation-SART = motivation in the sustained attention to response task; Motivation-0B = motivation in the 0-back task; Motivation-1B = motivation in the 1-back task; Interest-SART = interest in the sustained attention to response task; Interest-0B = interest in the 0-back task; Interest-1B = interest in the 1-back task; Omission error of SART = omission error in the sustained attention to response task; Error-0B = error in the 0-back task; Error-1B = error in the 1-back task. ***p* < 0.01, **p* < 0.05.

**Table S6** Proportions of participants with different responses in focus back effort and motivation.

|  | LFBE and HM | HFBE and LM | LFBE and LM | HFBE and HM |
| --- | --- | --- | --- | --- |
| SART | 10.62% | 20.77% | 16.43% | 52.17% |
| 0-back task | 16.43% | 11.11% | 18.36% | 54.11% |
| 1-back task | 8.21% | 16.91% | 13.04% | 61.84% |
| RCT | 5.56% | 5.56% | 6.94% | 81.94% |

Note. LFBE = low focus back effort (average score ≤ 3); HM = high motivation (score > 3); HFBE = high focus back effort (average score > 3); LM = low motivation (score ≤ 3); SART = sustained attention to response task; RCT = reading comprehension task.

**Table S7** Factor loadings for structural modeling models.

|  | | | MW | | | | FBE | | | Motivation | | | | Interest | | |
| --- | --- | --- | --- | --- | --- | --- | --- | --- | --- | --- | --- | --- | --- | --- | --- | --- |
|  | SART | 0B | | 1B | SART | 0B | | 1B | SART | | 0B | 1B | SART | | 0B | 1B |
| Model 1 | 0.92 | 0.84 | | 0.80 | 0.85 | 0.81 | | 0.84 | 0.89 | | 0.78 | 0.82 | 0.80 | | 0.77 | 0.82 |
| Model 2 | 0.93 | 0.84 | | 0.81 | 0.86 | 0.81 | | 0.84 | 0.89 | | 0.78 | 0.83 | 0.79 | | 0.78 | 0.84 |
| FS1 | 0.93 | 0.84 | | 0.81 | 0.86 | 0.81 | | 0.84 | 0.89 | | 0.78 | 0.83 | 0.79 | | 0.78 | 0.84 |

Note. All paths are statistically significant at the *p* < 0.05 level. MW = mind wandering; FBE = focus back effort; SART = sustained attention to response task; 0B = 0 back task; 1B = 1 back task; FS1 = the model of Figure S1.

**Table S8** Descriptive statistics for variables in Study 2.

| Measure | *M* | *SD* | Range | Skewness | Kurtosis |
| --- | --- | --- | --- | --- | --- |
| MW | 2.82 | 0.74 | 1.50-5 | 0.788 | 0.07 |
| FBE | 4.04 | 0.84 | 1.67-5.67 | -0.35 | -0.35 |
| Motivation | 4.85 | 1.19 | 2-6 | -0.91 | 0.06 |
| Interest | 3.30 | 0.94 | 2-6 | 0.50 | 0.32 |
| RC | 6.28 | 1.41 | 2-8 | -0.82 | 0.06 |

Note. MW = mind wandering; FBE = focus back effort; RC = reading comprehension.

**Table S9** Correlation coefficients for all measures in Study 2.

| Measure | FBE | Motivation | Interest | RC |
| --- | --- | --- | --- | --- |
| MW | -0.40** | -0.40** | -0.29** | -0.27** |
| FBE |  | 0.52** | 0.30** | 0.05 |
| Motivation |  |  | 0.40** | 0.25** |
| Interest |  |  |  | 0.23** |

Note. MW = mind wandering; FBE = focus back effort; RC = reading comprehension. ***p* < 0.01.

**Table S10** Fixed effects of the multilevel logistic regression model (Model S4): focus back effort in the reading comprehension. task.

|  | Estimate | *SE* | *z* | *p* | Odds ratio | 95% CI |
| --- | --- | --- | --- | --- | --- | --- |
| (Intercept) | 4.41 | 0.10 | 42.15 | < 0.001 | 81.90 | (66.71, 100.58) |
| Trial | -0.13 | 0.03 | -4.89 | < 0.001 | 0.87 | (0.83, 0.92) |

Note. Confidence intervals of 95% are for the odds ratio. This model was based on 717 observations.**
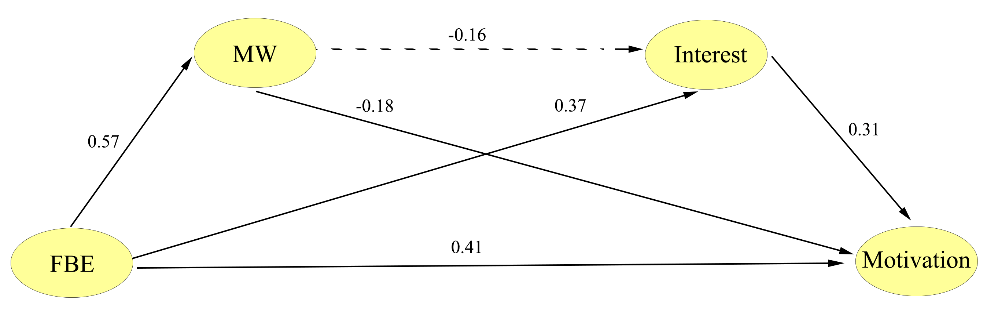
**

**Fig. S1** Mediation effect paths of mind wandering (MW) and interest between focus back effort (FBE) and motivation. The single-headed arrow from one latent variable (circle) to another latent variable represents the contribution of each latent variable to the other. All numbers appearing beside each arrow are standardized. Solid paths indicate significant relationships at *p* < 0.05, while dotted lines indicate non-significant relationships at *p* < 0.05. To simplify the interpretation process, the factor loadings of manifest variables have been excluded from the illustration and could be found in Table S7.


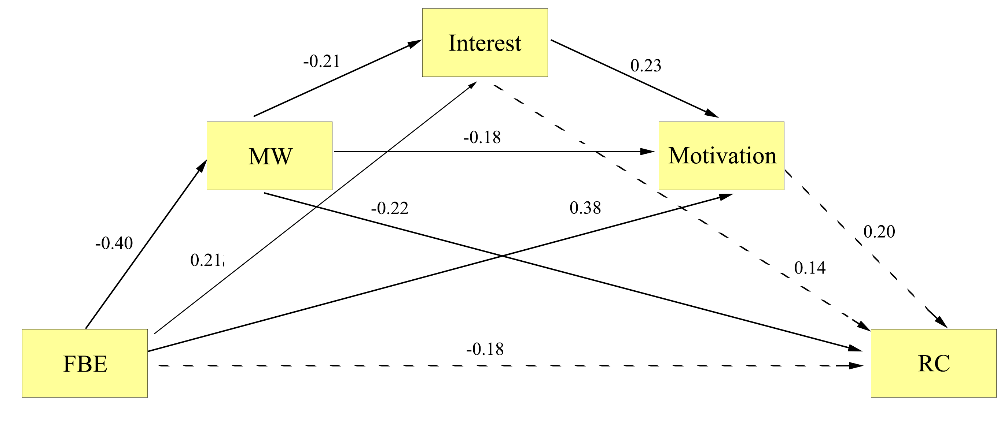


**Fig. S2** Mediation effect paths of mind wandering (MW), interest, and motivation between focus back effort (FBE) and reading comprehension (RC) in Study 2 (Model 6). The single-headed arrow from one (square) to another variable represents each variable’s effect on one another. All numbers appearing beside each arrow are standardized. Solid paths indicate significant relationships at *p* < 0.05, while dotted lines indicate non-significant relationships at *p* < 0.05.

**References**

Kline, R. B. (1998). *Principles and practice of structural equation modeling*. New York: Guilford Press.

Kuznetsova, A., Brockhoff, P. B., & Christensen, R. H. (2017). lmerTest package: Tests in linear mixed effects models. *Journal of Statistical Software*, *82*(13), 1-26.

Marcusson-Clavertz, D., West, M., Kjell, O. N. E., & Somer, E. (2019). A daily diary study on maladaptive daydreaming, mind wandering, and sleep disturbances: Examining within-person and between-persons relations. *Plos One*, *14*(11).

Seli, P., Wammes, J. D., Risko, E. F., & Smilek, D. (2016). On the relation between motivation and retention in educational contexts: The role of intentional and unintentional mind wandering. *Psychonomic Bulletin & Review*, *23*(4), 1280-1287.

Smallwood, J., & Schooler, J. W. (2006). The restless mind. *Psychological Bulletin*, *132*(6), 946-958.

Thomson, D. R., Besner, D., & Smilek, D. (2015). A resource-control account of sustained attention: Evidence from mind-wandering and vigilance paradigms. *Perspectives on Psychological Science*, *10*(1), 82-96.
