# Supplementary figures and images for: The role of focus back effort in the relationships among motivation, interest, and mind wandering: an individual difference perspective
Source: Cogn Res Princ Implic. 2023 Jul 13;8:43. doi: 10.1186/s41235-023-00502-0 (PMC10344852; doi:10.1186/s41235-023-00502-0)

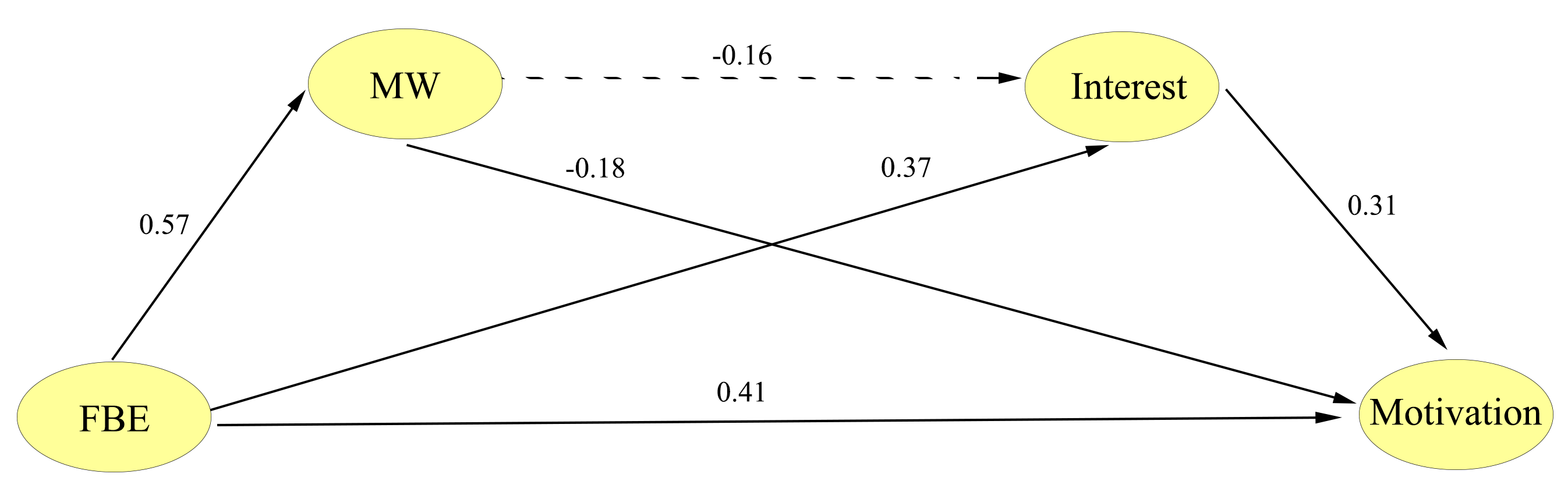

Supplement: Supplementary file 2 — Additional file 2. Mediation effect paths of mind wandering and interest between focus back effort and motivation in Study 1. [file 41235_2023_502_MOESM2_ESM.tif]

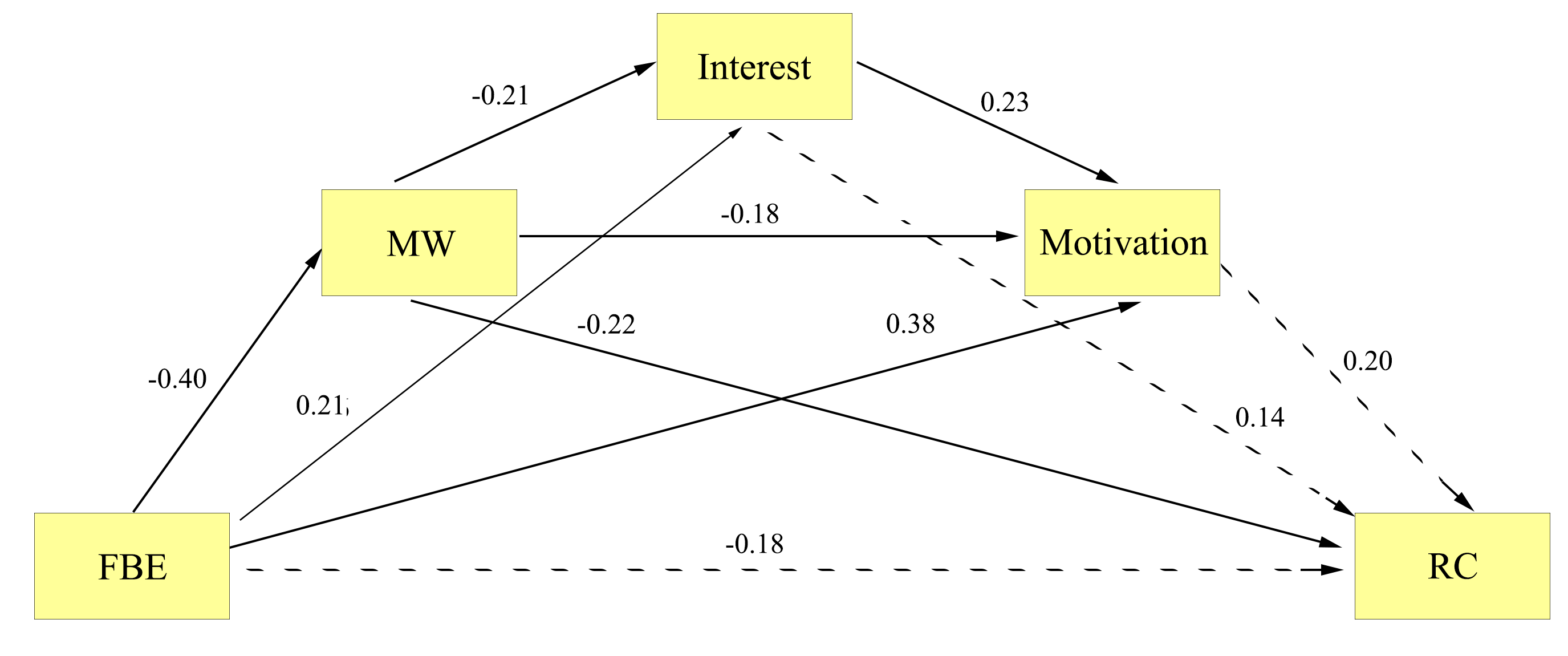

Supplement: Supplementary file 3 — Additional file 3. Mediation effect paths of mind wandering , interest, and motivation between focus back effort and reading comprehension in Study 2. [file 41235_2023_502_MOESM3_ESM.tif]
